# Ultrasound-assisted and resin-based purification of bioactive polyphenols from peony (*Paeonia ostii*) pods: process optimization and α-glucosidase inhibitory activity^☆^

**DOI:** 10.1016/j.ultsonch.2026.107775

**Published:** 2026-02-12

**Authors:** Xiaoai Zhu, Yuan Ru, Kebing Yan, Siqi Zhang, Yingjie Sun, Fengke Du, Yage Liu, Yiying Niu, Jun Xi, Kunlun Liu

**Affiliations:** a Food Engineering Technology Research Center/Key Laboratory of Henan Province, College of Food Science and Engineering, Henan University of Technology, Zhengzhou 450001, PR China; b Key Laboratory of Biology and Genetic Improvement of Oil Crops, Key Laboratory of Oilseeds Processing, Ministry of Agriculture and Rural Affairs, Oil Crops Research Institute, Chinese Academy of Agricultural Sciences, Wuhan 430062, PR China

**Keywords:** Bioactivity, Peony pods, Phenolic compounds, Ultrasound-assisted extraction, UPLC–MS/MS

## Abstract

The valorization of agricultural by-products into high-value ingredients requires efficient and green extraction technologies. Ultrasonic-assisted extraction (UAE) has emerged as a promising technique for this purpose due to its efficiency and environmental benefits. In this study, an integrated green process was developed for recovering bioactive polyphenols from peony (*Paeonia ostii*) pods (PPP), an underutilized by-product, using combined UAE and macroporous resin purification. The ultrasonication process was systematically optimized via response surface methodology. The determined optimal conditions (46 % ethanol, 14 mL/g liquid–solid ratio, 60 min ultrasonication) achieved a yield of PPP of 52.17 ± 0.06 mg GAE/g DW. Subsequent purification employing D101 macroporous resin and 40 % ethanol elution produced a refined polyphenol fraction, PPP40, with a purity of 43.93 %. Untargeted metabolomic profiling revealed 17 major phenolic constituents in PPP40, including gallic acid, kaempferol 7-*O*-glucoside, isorhamnetin-3-glucoside-4′-glucoside, and ethyl gallate as predominant compounds. The functional efficacy of the purified PPP40 fraction was evaluated based on its α-glucosidase inhibitory activity. PPP40 exhibited potent inhibition, with an IC_50_ value of 639.96 ± 4.57 μg/mL, and acted via a mixed-type inhibition mechanism. Multi-spectroscopic analyses elucidated that the inhibitory mechanism involved dynamic fluorescence quenching and concomitant conformational changes in α-glucosidase. The proposed integrated ultrasound-resin process offered an efficient and sustainable strategy for valorizing agricultural by-products, yielding a well-characterized and polyphenol-enriched fraction with potential application as a functional food ingredient for regulating postprandial blood glucose management.

## Graphical abstract

From waste to bioactives: α-Glucosidase inhibition by polyphenols from peony pods through multimethod analysis.

**Figure:**
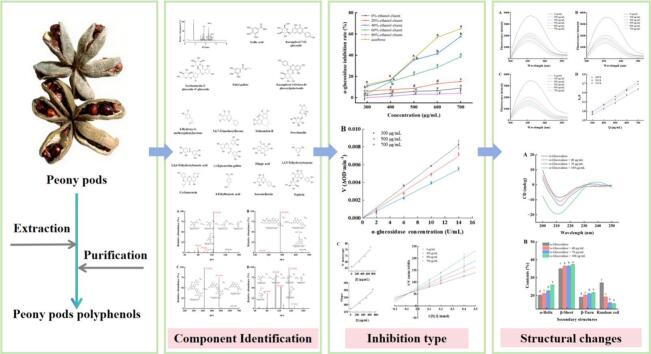

## 1 Introduction

Diabetes mellitus, characterized by metabolic abnormalities and multiple systemic complications, is globally recognized as a major chronic disease [1]. The number of individuals with diabetes worldwide is expected to rise to approximately 853 million by the year 2050 [2]. Among diabetic cases, type 2 diabetes mellitus (T2DM) is the predominant type, representing more than 90 % of the total confirmed diagnoses. At present, the preferred drugs for T2DM include insulin and metformin. However, metformin is often associated with gastrointestinal side effects, while insulin therapy carries a significant risk of hypoglycemia and weight gain. Critically, neither intervention arrests the progressive decline in β-cell function inherent to the disease, and long-term exogenous insulin administration may itself contribute to worsening peripheral insulin resistance [3]. Consequently, the identification of safe and efficacious natural alternatives for the prevention and treatment of T2DM has become a critical focus of research work.

Polyphenols are a group of bioactive compounds of great diversity found ubiquitously among plants [4], [5]. Polyphenols have multiple health benefits, including antioxidant, antidiabetic, and antimicrobial activities [6], [7], [8], [9]. Among these, polyphenolic compounds exert their antioxidant activity primarily by scavenging free radicals [10], [11]. In the case of T2DM management, polyphenols have been proven effective significantly in the prevention as well as treatment of the disease by inhibiting the catalytic activities of α-amylase and α-glucosidase [12], [13], [14]. The inhibitory process prevents the breakdown of carbohydrates into glucose units, thereby significantly preventing postprandial hyperglycemia. The aforementioned observations have been evidenced by the related studies, e.g., the report by Parmenter et al. [15] revealed a causal relationship between sustained dietary intake of flavonoids and a reduction in T2DM incidence, with a potential decrease of approximately 20 %. Thus, the research work on polyphenolic α-glucosidase inhibitors from plants has great theoretical importance as well as practical worth for the management and cure of T2DM.

After National Health Commission approval for peony seed oil to be sold on the food market, the industry has grown dramatically [8]. Nevertheless, sustainable exploitation has been set back considerably by the wasteful exploitation of peony pods, representing some 60 % of the overall peony fruit mass [16]. The pods are rich in bioactive compounds, the polyphenolic compounds representing around 18.65–19.92 % of the overall composition [17]. The key polyphenolic compounds found by Jin et al. [18] in peony pods are gallic acid, methyl gallate, and quercetin. Additionally, Bai et al. [19] reported phenolic constituents such as paeoniflorin and methyl gallate in these pods, attributing significant antioxidant and antibacterial activities to these substances. These polyphenols exhibit notable bioactivities; however, research focusing on peony pods remains preliminary. Most existing studies have prioritized the identification of phenolic constituents [19] or the bioactivity screening of crude extracts [18], while systematic investigations into process-optimized extraction and the mechanistic basis of their α-glucosidase inhibition are notably scarce, thereby constraining their practical application.

Effective extraction and purification of polyphenols is a fundamental prerequisite for reliable bioactivity evaluation [20]. UAE has emerged as a significant green technology in the field of phytochemistry, particularly suitable for extracting antioxidants from plant matrices [21]. Its effectiveness stems from acoustic cavitation, which generates intense shear forces and microturbulence, mechanistically leading to cell wall rupture, enhanced solvent penetration, and improved mass transfer [22], [23], [24]. Recent studies have highlighted its practical advantages in extracting heat-sensitive polyphenols: significantly reducing extraction time, minimizing solvent usage, and minimizing thermal degradation, thereby preserving the integrity of bioactivity [25], [26]. For dense agricultural by-products, UAE demonstrates superior efficiency in disrupting rigid structures and releasing bound phenolic compounds compared to traditional methods [27]. Post-extraction, macroporous resin purification is often employed to achieve selective enrichment. This step utilizes hydrophobic interactions and hydrogen bonding to adsorb target polyphenols, effectively removing sugars, proteins, and other polar impurities [28]. Therefore, this study synergistically combines the efficiency of UAE with the selective purification of macroporous resin, aiming not only to efficiently recover polyphenols from peony pods by-products but also to prepare comprehensively characterized components for subsequent in-depth research on their bioactivity and mechanisms.

This study was designed to develop an integrated strategy for the valorization of peony pod by-products. This involved: (1) optimizing the UAE process of PPP using response surface methodology (RSM) and further purifying them with macroporous adsorption resin (MAR); (2) identifying the primary constituents of the obtained PPP through untargeted metabolomics analysis by employing ultra-performance liquid chromatography coupled with tandem mass spectrometry (UPLC–MS/MS); and (3) evaluating the antioxidant potential and elucidating the mechanism of α-glucosidase inhibition via enzyme kinetics, fluorescence quenching, and CD spectroscopy. Employing these systematic methodologies enabled the efficient extraction and comprehensive profiling of PPP, confirming its dual roles as an effective antioxidant and α-glucosidase inhibitor. These results collectively highlighted the significant potential of PPP as a functional food ingredient for regulating postprandial blood glucose levels.

## 2 Materials and methods

### 2.1 Materials and chemicals

Peony pods (*Paeonia ostii* ‘Fengdan’) were collected from Yangshan, Song County, Luoyang, Henan, China. Plants were grown in open fields with compound fertilizer and drip irrigation as needed. Mature pods were manually collected from 100 healthy, genetically distinct plants to ensure representativeness. Samples were transported immediately to the lab, dried at 50 °C to constant (moisture content ≤2 %) using an FXB 101-1 oven (Shu Li, China), milled, and sieved through a 60-mesh stainless-steel screen. The powder was stored at −20 °C in nitrogen-flushed, airtight bags and used within 30 days. Folin phenol reagent, anhydrous sodium carbonate, gallic acid, α‑glucosidase, DPPH, ABTS, p‑nitrophenyl‑α‑D‑glucopyranoside (pNPG), X‑5, D101, AB‑8, and NKA9 MAR were obtained from Yuanye Biotechnology Co., Ltd. (Shanghai, China). Hydrochloric acid was purchased from Xilong Science Co., Ltd. (Shantou, China), while anhydrous ethanol, sodium dihydrogen phosphate, disodium hydrogen phosphate, and potassium persulfate were procured from Kemiou Chemical Reagent Co., Ltd (Tianjin, China). Methanol was procured from Merck KGaA (Darmstadt, Germany). Acetonitrile was procured from Xingke High Purity Solvent Co., Ltd. (Shanghai, China). Formic acid was procured from Aladdin Biochemical Technology Co., Ltd. (Shanghai, China).

### 2.2 Ultrasound-assisted extraction (UAE) method

PPP were extracted via ultrasound‑assisted ethanol extraction. Single-factor experiments were conducted to investigate the effects of ethanol concentration, liquid-to-solid ratio, and ultrasonication time on PPP extraction yield. Briefly, 1 g of powdered peony pods was placed in 50 mL centrifuge tubes with ethanol solutions (0, 15, 30, 45, 60, 75, and 90 %) at liquid–solid ratios of (5, 10, 15, 20, 25, 30, 35 mL/g). After vortexing for 10 s to ensure homogeneity, the extract underwent sonication in an ultrasonic cleaner (GA008C, Granbo Technology, Shenzhen, China) for designated durations (0–120 min), with periodic vortexing interruptions. After ultrasonic extraction, the mixture was centrifuged at 3500 rpm for 10 min in a centrifuge (TDL-5A, Jintan Medical Instrument Factory, Jiangsu, China). Supernatants were collected, and the procedure was repeated once. The combined supernatants were then adjusted to 50 mL for subsequent analysis. Subsequent response surface experiments were conducted based on the results of the single-factor experiments.

### 2.3 Extraction process optimization based on RSM

The strength of RSM lies in its capacity to minimize the number of experiments required to evaluate variables and their interactions [29]. Initial extraction conditions were optimized using single‑factor experiments. A Box–Behnken design (BBD) was subsequently applied to investigate the combined effects of three independent factors at three levels. Independent variables were ethanol concentration (A), liquid–solid ratio (B), and extraction time (C), with PPP content of extract as the dependent variable. Details in Table S1.

Design-Expert 13 software built the BBD and response surface models, and ANOVA assessed their viability. Ultimately, a quadratic regression model was used to model the relationship between experimental factors and the response variable:

(1) $$Y=\beta_{0}+\sum_{j=1}^{k}\beta_{j}X_{j}+\sum_{j=1}^{k}\beta_{\mathrm{jj}}X_{j}^{2}+\sum_{i<j}\beta_{\mathrm{jj}}X_{i}X_{j}\left(k=3\right)$$

where Y represents the response; X_i_ and X_j_ denote the independent variables; β_0_ is the constant coefficient; β_j_, β_jj_, and β_ij_ represent the coefficients of the linear, squared, and interaction terms, respectively; and k signifies the number of factors.

### 2.4 Total polyphenol content (TPC) determination

The TPC assay was conducted using a slightly adapted method from Zhou et al. [30]. Briefly, 0.5 mL of sample or control was pipetted into tubes. Then, 2.5 mL of Folin–Ciocalteu reagent (0.1 mol/L) was added to each tube, followed by 2 mL of sodium carbonate (7.5 %, w/v) solution. All samples were then incubated at 25 °C in darkness for 60 min. The absorbance was measured at a wavelength of 765 nm using a UV–1700 spectrophotometer (Shimadzu Corporation, Kyoto, Japan). TPC was calculated using a standard calibration curve (Y = 0.005X + 0.0195, R^2^ = 0.9959) generated with gallic acid standards ranging from 0 to 140 μg/mL. Results were expressed as mg gallic acid equivalents (GAE) per gram of dry matter, as calculated by Eq. (2):

(2) TPC(mg/g) =(C×V×N)/m

where C denotes gallic acid polyphenol concentration (μg/mL), V represents sample volume (mL), N stands for the dilution factor, and m indicates peony pods powder mass (g).

### 2.5 Purification of PPP using macroporous adsorption resin (MAR)

#### 2.5.1 Pretreatment of MAR

Before adsorption experiments, the MARs were pretreated. The MARs were first treated by soaking them in absolute ethanol for 24 h, and then washed with deionized water to exclude the residual ethanol. After that, the MARs were treated successively by soaking them in 5 % HCl solution (v/v) for 4 h, followed by incubation with 5 % NaOH solution (w/v) for 4 h, before they were washed with deionized water until the solution was neutral.

#### 2.5.2 Screening of MARs

Four MARs (AB-8, X-5, D101, and NKA-9) were evaluated for their adsorption/desorption efficiency to determine optimal MARs for PPP purification following Shen et al.'s [31] methodology. MARs (3 g wet weight) were combined with 25 mL PPP solution in 150 mL flasks and incubated at 25 °C, 120 rpm for 12 h. The concentration of PPP adsorbed in solution (C_1_) was determined by spectrophotometry. We calculated the adsorption capacity and adsorption rate (%) using Eqs. (3), (4). MARs were filtered, washed with 300 mL deionized water, shifted into new conical flasks, and desorbed using 50 mL ethanol (95 % v/v) at 25 °C for 12 h with agitation at 120 rpm. The concentration of the PPP adsorbed by spectrophotometry was quantitated (C_2_). Desorption capacity was also calculated by Eqs. (5), (6) alongside the calculation for the desorption rate (%).

(3) $$Adsorptioncapacity\left(\mathrm{mgGAE}/gDW\right)=\frac{\left(C_{0}-C_{1}\right)\times V_{1}}{M}$$

(4) $$\mathrm{Adsorptionrate}=\frac{\left(C_{0}-C_{1}\right)}{C_{0}}\times 100\%$$

where C_0_ is initial polyphenol concentration (mg GAE/mL); C_1_ is polyphenol concentration in breakthrough solution postadsorption (mg GAE/mL); and M is the MAR mass (g).

(5) $$Desorptioncapacity\left(\mathrm{mgGAE}/gDW\right)=\frac{C_{2}V_{2}}{M}$$

(6) $$\mathrm{Desorptionrate}=\frac{C_{2}V_{2}}{\left(C_{0}-C_{1}\right)\times V_{1}}\times 100\%$$

where C_2_ is polyphenol concentration in eluent (mg GAE/mL); V_1_ is volume of crude extract (mL); V_2_ is volume of eluent (mL).

#### 2.5.3 Static adsorption and desorption kinetics

Pretreated D101 MAR (3 g wet weight) was placed in a 150 mL conical flask and equilibrated with 50 mL of PPP crude extract (1 mg GAE/mL). The system was agitated at 120 rpm and maintained at 25 °C. Samples were collected every 30 min for 6 h to assess PPP concentration, facilitating static adsorption kinetic modeling for D101 MAR. After adsorption equilibrium, the MAR was cleaned with pure distillate until the outflow was clear. Desorption was performed with 50 mL ethanol (95 % v/v) at 120 rpm, and the PPP concentration in eluent was measured at 30 min intervals to establish desorption kinetic profiles. For assessing the adsorption kinetics, the acquired data were accommodated with two prevalent kinetic models: the pseudo-first-order (PFO) (7) and pseudo-second-order (PSO) models (8):

(7) $$\ln\left(Q_{e}-Q_{t}\right)=-k_{1}t+\ln\left(Q_{e}\right)$$

(8) $$\frac{t}{Q_{t}}=\frac{1}{k_{2}Q_{e}^{2}}+\frac{t}{Q_{e}}$$

where Q_e_ (mg GAE/g DW) represents the adsorption capacity at equilibrium; Q_t_ (mg GAE/g DW) corresponds to the adsorption capacity at a given time t (min); and the rate constants are labeled as k_1_ (min^−1^) for the PFO model and k_2_ (g/(mg·min)) for the PSO model.

#### 2.5.4 Static adsorption assay

In a separate procedure, pretreated D101 MAR (3 g wet weight) was added to 150 mL conical flasks containing 25 mL of PPP solutions at gradient concentrations of gallic acid equivalents (0.5, 1.0, 1.5, 2.0, and 2.5 mg/mL). The mixtures were agitated at 120 rpm for 6 h at 25 °C until equilibrium adsorption was attained. After adsorption, PPP concentrations in the solutions were quantified spectrophotometrically, and the adsorption rates were computed using formulas (2) and (4).

Similarly, pretreated D101 MAR (3 g wet weight) was placed into 150 mL conical flasks, each containing 25 mL PPP solution (1.5 mg GAE/mL) at different pH levels. The mixtures were shaken at 120 rpm for 6 h at 25 °C in a water bath shaker to achieve adsorption equilibrium. After reaching equilibrium, PPP concentration remaining in the supernatants was determined spectrophotometrically, and the adsorption efficiency was calculated according to Eqs. (2), (4).

#### 2.5.5 Gradient elution profiling

Ethanol–water solutions of varying concentrations were prepared in 20 % increments, with each volume corresponding to two bed volumes (BVs). A glass column (20 × 300 mm) was packed with 110 mL of pretreated D101 MAR. After equilibrating the column with deionized water (2 BVs), 3 BVs of the PPP extract were loaded at 1 BV/h. Once saturation was achieved, gradient elution was performed at 1 BV/h using the ethanol gradient sequentially. Fractions of 50 mL were collected, and the PPP concentration was determined spectrophotometrically at 765 nm. An elution profile was plotted with elution volume (mL) as the abscissa and the PPP concentration (mg GAE/mL) as the ordinate. The optimal eluent concentration was determined based on peak resolution and the PPP recovery rate in the elution fractions.

### 2.6 Untargeted metabolomics analysis by UPLC–MS/MS

#### 2.6.1 Sample preparation

Fractions of PPP eluted with 40 % ethanol were freeze-dried using a Scientz-100F lyophilizer (Shun Zhi, China) and then finely ground at 30 Hz for 1.5 min using an MM 400 grinder (Retsch, Germany). Approximately 50 mg of powdered sample was mixed with 1200 μL of 70 % methanol solution pre-cooled to –20 °C. The mixture was vortexed for 30 s at 30 min intervals over 180 min. After centrifugation at 12,000 rpm for 3 min at 4 °C, the supernatants were filtered through 0.22 μm nylon membranes (Millipore) and stored at –80 °C for further analysis.

#### 2.6.2 Chromatography and mass spectrometry acquisition conditions

Chromatographic separation was conducted on an ACQUITY UPLC HSS T3 column at 40 °C and a flow rate of 0.4 mL/min. The mobile phase consisted of (A) water containing 0.1 % formic acid and (B) acetonitrile containing 0.1 % formic acid. An injection volume of 4 μL was used. The UPLC gradient was programmed as follows: starting at 95 % A and 5 % B, changing to 35 % A and 65 % B at 5.0 min; then adjusted to 1 % A and 99 % B at 6.0 min and held until 7.5 min; finally, returning to 95 % A and 5 % B at 7.6 min and maintaining this composition until 10.0 min.

Mass spectrometric detection was performed on a TripleTOF 6600 system (Sciex, USA) under the following conditions: ionization mode ESI ±; curtain gas 35 psi; GS1 50 psi; GS2 60 psi; ion spray voltage +5500 V (positive) and –4500 V (negative); source temperature 550 °C; MS1 scan range *m*/*z* 50–1250, CE 10 V; MS2 scan range *m*/*z* 25–1250, CE 30 V.

#### 2.6.3 Qualitative and quantitative analysis of components

The raw data were transformed into mzXML (ProteoWizard 3.0) and analyzed using XCMS (v3.8) for peak detection, alignment, and retention time (RT) adjustment. Variables with over 50 % missing data were excluded, and the remaining gaps were filled using K-nearest neighbor imputation. Normalization of peak areas was performed using support vector regression (SVR) based on QC samples. The processed peaks underwent metabolite identification utilizing the laboratory's proprietary database, public repositories, library matching, and the metDNA technique. Compounds with a composite score >0.5 and CV value <0.5 in the QC samples were isolated and merged into positive and negative extracts.

### 2.7 Free radical scavenging assay

DPPH and ABTS scavenging capacity served as initial assessments of PPP's antioxidant potential. Vitamin C (V_C_) solution was used as the positive control. DPPH radical scavenging capacity was assessed using a modified protocol from Altarjami et al. [9]. 1 mL of PPP solutions were mixed with 5 mL of DPPH solution, vortexed, then incubated at 25 °C for 45 min before measuring absorbance at 517 nm. The ABTS radical scavenging assay was conducted according to the method described by Le Tan et al. [32], with minor modifications. 0.4 mL of various PPP concentrations was mixed with 3.6 mL of ABTS working solution, incubated for 6 min at 25 °C in the dark, and absorbance measured at 734 nm.

### 2.8 Inhibitory effect and mechanism of PPP on α-glucosidase

#### 2.8.1 Inhibitory activity of PPP against α-glucosidase

Determination of PPP's α-glucosidase inhibition was carried out according to a modified procedure by Maibam et al. [33]. Acarbose was used as a positive control. 0.5 mL of the solution of PPP or acarbose solutions was incubated with 1 mL solution of the α-glucosidase (10 U/mL) at 37 °C for 10 min. After adding 1 mL solution of pNPG (5 mmol/L), mixtures incubated for 50 min at 37 °C. The reaction terminated with Na_2_CO_3_, followed by a 10-min incubation. Absorbance was recorded at 405 nm. Inhibition percentage was calculated using the following Eq. (9):

(9) $$\text{Inhibition rate}\;\left(\%\right)=\left[\text{1}-\frac{A_{1}-A_{2}}{A_{3}-A_{4}}\right]\times \text{1}00\%$$

where A_1_, A_2_, A_3_ and A_4_ represent the absorbances in the presence of inhibitor and α-glucosidase, in the absence of α-glucosidase, in the absence of inhibitor, and in the absence of inhibitor and α-glucosidase, respectively.

#### 2.8.2 Inhibition type and inhibition constants of PPP

The study utilized Xu et al.'s [34] method to measure kinetic inhibition, evaluating reaction reversibility by initial rate assessments at various enzyme concentrations. Reaction rates were assessed for PPP concentrations (300, 500, 700 μg/mL) across α-glucosidase levels (0, 2, 6, 10, 14 U/mL) with a 5 mmol/L pNPG substrate. Absorbance data determined the reaction rate, plotted against enzyme concentration to assess inhibition reversibility.

According to the method of Song et al. [35], Lineweaver–Burk plots were used to evaluate the inhibition of α-glucosidase by diverse inhibitors. The α-glucosidase concentration was maintained at 10 U/mL, with polyphenol concentrations (300, 500, 700 μg/mL) evaluated against pNPG at various (0, 2, 6, 10, 14 U/mL) levels. The absorbance was measured for double inverse plotting. The V_max_, K_m_, K_i_ and K_is_ values were calculated using the following Eqs. (10), (11), (12):

(10) $$\frac{1}{V}=\frac{K_{\text{m}}}{V_{\max}}\left(1+\frac{\left[I\right]}{K_{i}}\right)\frac{1}{\left[S\right]}+\frac{1}{V_{\max}}\left(1+\frac{\left[I\right]}{K_{\text{is}}}\right)$$

(11) $$\mathrm{Slope}=\frac{K_{m}}{V_{\max}}\frac{\left[I\right]}{K_{i}}+\frac{K_{m}}{V_{\max}}$$

(12) $$Y-Intercept=\frac{1}{V_{\max}}\frac{\left[I\right]}{K_{\mathrm{is}}}+\frac{1}{V_{\max}}$$

where [I] and [S] denote the levels of PPP and substrate (pNPG), respectively; K_m_ and V_max_ denote the enzyme's affinity (Michaelis–Menten constant) and peak velocity (maximum reaction rate) for α-glucosidase; K_i_ signifies the binding affinity for α-glucosidase–PPP, while K_is_ represents the binding affinity for α-glucosidase–pNPG.

#### 2.8.3 Fluorescence quenching spectroscopy

The fluorescence quenching experiments were conducted using a modified method from Xing et al. [36]. Each reaction mixture contained 3 mL α-glucosidase, 0.5 mL PPP solution, and 1 mL 0.2 M PBS (pH 6.8), vortexed for 30 s, then equilibrated for 60 min at 298, 304, or 310 K. Fluorescence spectra between 300–400 nm were recorded using a fluorescence spectrophotometer (G9800A, Agilent Technologies, USA) at an excitation wavelength of 280 nm, with excitation and emission monochromator slit widths of 5.0 nm. PBS buffer was used as a blank. Stern-Volmer kinetics Equation (13) was used to evaluate the quenching mechanism. Thermodynamic parameters, such as enthalpy change (ΔH) and entropy change (ΔS), were derived using the Van't Hoff Equation (14), while Gibbs free energy change (ΔG) was subsequently calculated through the Gibbs–Helmholtz Equation (15).

(13) $$\frac{F_{0}}{F}=1+K_{\text{q}}\tau \left[Q\right]=1+K_{\mathrm{SV}}\left[Q\right]$$

(14) $$\log K_{\mathrm{SV}}=-\frac{\Delta H}{2.303R}\frac{1}{T}+\frac{\Delta S}{2.303R}$$

(15) ΔG=ΔH-TΔS

where F_0_ represents fluorescence intensity without inhibitor; F denotes fluorescence intensity with inhibitor; τ_0_ is the fluorescence lifetime of free enzyme (ns); K_SV_ and K_q_ represent Stern–Volmer quenching constant and the bimolecular quenching constant, respectively; [Q] represents the inhibitor concentration; R represents the gas constant, equal to (8.314 J·mol^−1^·K^−1^), and T indicates temperature in kelvins (K).

#### 2.8.4 Circular dichroism (CD)

α-Glucosidase solution (0.3 mg/mL) was mixed with PPP in a 1:2 M ratio in 0.2 M PBS (pH 6.8) at 37 °C for 20 min. Reference spectra were acquired using 0.2 M PBS (pH 6.8) for baseline subtraction. Samples were loaded into 1.0 mm path length quartz cuvettes, and circular dichroism spectra were recorded from 200 to 250 nm using a CD spectroscopy (Jasco Corp J 1500, Tokyo, Japan). Secondary structure elements proportions were determined using CDNN deconvolution software on CD data.

### 2.9 Statistical analysis

All experiments were repeated three times, with data were presented as the mean ± standard deviation (SD). Statistical analyses were performed using IBM SPSS Statistics 27 software. Differences among groups were evaluated by one-way ANOVA with subsequent Duncan's multiple comparison test, establishing statistical significance at P < 0.05. Data were statistically visualized using Origin 2024 software.

## 3 Results and discussion

### 3.1 Optimization of the ultrasound–assisted ethanol extraction method

#### 3.1.1 Results of the single factor assay

**Fig. 1:**
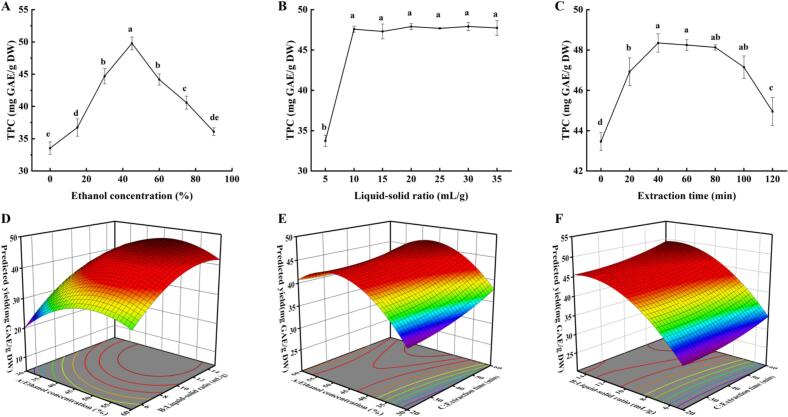
Single–factor extraction experimental results: ethanol concentration (A), liquid–solid ratio (B), and extraction time (C). Response surface analysis results: effect of ethanol concentration and liquid–solid ratio (D); effect of ethanol concentration and extraction time (E); effect of liquid–solid ratio and extraction time (F). Results are presented as mean ± standard deviation (n = 3). Different lowercase letters in the line graph indicate significant differences (P < 0.05).

Fig. 1A illustrated a parabolic relationship between ethanol concentration and PPP content of extract, peaking at 45 % ethanol (49.79 ± 0.99 mg GAE/g DW). Ethanol boosted PPP extraction efficiency by improving solubility and membrane permeability, thereby improving the extraction efficiency by enhancing the concentration gradient [37]. Beyond 45 % ethanol, hydrophobic compound co-extraction decreased PPP solubility in the binary solvent system [38]. Thus, 30 %, 45 %, and 60 % ethanol were selected for RSM optimization.

In Fig. 1B, a notable increase in PPP content of extract was observed with a liquid‒solid ratio increasing from 5 to 10 mL/g, reaching a plateau at 10 mL/g with a value of 47.59 ± 0.38 mg GAE/g DW. The stabilization of PPP content of extract beyond 10 mL/g indicated solvent saturation for PPP dissolution [39]. Consequently, ratios of 5, 10, and 15 mL/g were selected for further investigation.

Fig. 1C demonstrated the time-dependent PPP extraction profile. The yield increased linearly to 48.35 ± 0.46 mg GAE/g DW at 40 min, following which it gradually declined with prolonged extraction time. This decline corresponded to extraction equilibrium, indicating that mass transfer could not proceed further [40]. Extended extraction periods promoted competitive hydrophobic dissolution, reducing PPP content of extract [37]. Consequently, time durations of 20, 40, and 60 min were chosen for RSM optimization.

#### 3.1.2 Response surface fitting results

RSM experiment was designed based on the single-factor outcomes. The response surface experimental design and results were shown in Table S2. A multivariate regression analysis established the relationship between PPP content of extract and crucial extraction parameters, presented in Eq. (16):

(16) $$Y=43.71+3.16A+7.68B+1.3C-1.28AB-1.01AC+0.18BC-64A^{2}-5.08B^{2}+1.0C^{2}$$

where Y, A, B and C represent PPP content of extract (mg GAE/g DW), ethanol concentration (%), liquid‒solid ratio (mL/g), and extraction time (min), respectively.

ANOVA was utilized to evaluate the significance of regression parameters, and the outcomes were detailed in Table 1. A coefficient was deemed statistically significant if its P < 0.05. The model exhibited high statistical significance (P < 0.0001), whereas the lack of fit was nonsignificant (P = 0.6262, P > 0.05), confirming the model's adequacy [41]. Both R^2^ (0.9964) and adjusted R^2^ (R^2^_adj_ = 0.9919) approached 1, with a negligible difference (<0.2) between R^2^_adj_ and the predicted R^2^ (R^2^_pre_ = 0.9777), indicating a robust model with high predictive capability for PPP content of extract [40], [42]. The low coefficient of variation (CV = 81.73 %; <10 %) further confirmed the experiment's reproducibility and precision. All three factors' linear and quadratic terms significantly influenced the PPP content of extract. Interaction effects were significant for AB and AC but not for BC. Based on F-values, the factors ranked by influence were: liquid‒solid ratio > ethanol concentration > extraction time. This order differed from some studies on pomegranate peel, where ethanol concentration was the most influential factor [43], possibly due to the denser structure of peony pods requiring sufficient solvent volume for effective penetration. Conversely, it aligned with optimizations for of *Capparis spin* leaves [44], suggesting similarities in the mass transfer limitations of certain fruit by-products.

**Table 1:** ANOVA parameters and results.

Source	Sum of squares	Degree of freedom	Mean square	F-value	p-value	Significant level
Model	886.37	9	98.49	217.76	<0.0001	**
A	79.82	1	79.82	176.49	<0.0001	**
B	471.55	1	471.55	1042.63	<0.0001	**
C	23.84	1	23.84	52.71	0.0002	**
AB	6.50	1	6.50	14.38	0.0068	
AC	4.06	1	4.06	8.98	0.0200	*
BC	0.1296	1	0.1296	0.2866	0.6090	
A^2^	175.89	1	175.89	388.90	<0.0001	**
B^2^	108.48	1	108.48	239.85	<0.0001	**
C^2^	4.70	1	4.70	10.40	0.0146	*
Residual	3.17	7	0.4523			
Lack of Fit	1.03	3	0.3437	0.6439	0.6262	Not significant
Pure Error	2.13	4	0.5337			
Cor Total	889.54	16				

* Indicates a significant difference (P < 0.05).

** Indicates a highly significant difference (P < 0.01).

#### 3.1.3 Results of response surface analysis

Fig. 1D–F illustrated the interactive influence of ethanol concentration, liquid–solid ratio, and extraction time on PPP extraction efficiency. Fig. 1D revealed that higher ethanol concentration and liquid–solid ratio initially boosted PPP content of extract to a peak, followed by a minor decline. The optimal extraction conditions yielding the highest PPP concentration (48.9 mg GAE/g DW) were approximately 45–50 % ethanol with a liquid–solid ratio ranging between 13–14 mL/g. Fig. 1E revealed that ethanol concentration more significantly affected PPP extraction yield than extraction time at a constant liquid–solid ratio, as indicated by the contour plots. Fig. 1F indicates that liquid–solid ratio and extraction time exert minimal interactive influence on PPP content of extract remained relatively stable across varying extraction durations at both lower and higher liquid–solid ratios. These observations aligned well with earlier investigations by Feki et al. [29] and Khan et al. [45], which also explored optimization strategies for polyphenol extraction.

The RSM model determined the optimal extraction conditions (46 % ethanol, 14 mL/g liquid–solid ratio, 60 min ultrasonication) and predicted a corresponding maximum PPP content of 52.27 ± 0.13 mg GAE/g DW. For practical convenience, these conditions were slightly adjusted to ethanol concentration of 46 %, liquid–solid ratio of 14 mL/g, and extraction duration of 60 min. Triple verification test yielded 52.17 ± 0.06 mg GAE/g DW, demonstrating close agreement and confirming the accuracy and feasibility of the model. The final yield demonstrated a significant valorization potential, comparing favorably with yields reported for other notable agro-industrial by-products such as Capparis spinosa leaves (27.44 mg GAE/g) [44] and green tea (21.52–36.42 mg GAE/g DW) [45] under UAE, highlighting the promise of peony pods as a rich source of bioactive polyphenols.

### 3.2 Macroporous resin purification experiments

#### 3.2.1 Screening of MARs

Phytochemical enrichment performance depends on MAR's optimal adsorption and desorption capabilities, which correlate with physical properties including polarity, pore size, and specific surface area [46]. The best MAR for PPP enrichment was established by conducting four MARs having different physical qualities based on their capability for adsorption as well as their capability for desorption (Table 2). Indeed, the nonpolar resins X-5 and D101 had considerably higher PPP adsorption, depending on the “like dissolves like” principle [47]. Their large surface areas (>500 m^2^/g) provided abundant adsorption sites, enhancing van der Waals and π-π interactions [48]. Furthermore, smaller pore sizes improved adsorption efficiency by increasing contact area [49], with D101 featuring the smallest pores (9–10 nm). All four MARs displayed robust desorption capabilities, with D101 exhibiting the highest desorption rate. D101 was selected for PPP resin purification due to its superior adsorption, efficient desorption, and cost-effectiveness.

**Table 2:** Comparison of static adsorption–desorption performance of four types MARs.

Resins	Polarity	Pore diameter (nm)	Surface area (m^2^/g)	Adsorbing capacity (mg/g)	Adsorption rate (%)	Desorption capacity (mg/g)	Desorption rate (%)
X-5	Non-polar	28–0	500–600	10.42 ± 0.11^a^	82.94 ± 0.86^a^	9.94 ± 1.28^a^	95.21 ± 0.74^a^
D101	Non-polar	9–10	500–550	10.32 ± 0.02^ab^	82.09 ± 0.18^ab^	9.92 ± 0.24^a^	96.18 ± 2.13^a^
AB-8	Weakly polar	13–14	480–520	10.11 ± 0.03^b^	80.48 ± 0.20^b^	9.46 ± 0.29^ab^	93.49 ± 2.75^a^
NKA-9	Polar	10–12	170–250	9.73 ± 0.15^c^	77.42 ± 1.20^c^	9.29 ± 0.15^b^	95.51 ± 1.34^a^

Different lowercase letters represent significant differences among samples (p < 0.05).

#### 3.2.2 Static adsorption and desorption kinetics of D101 MAR

**Fig. 2:**
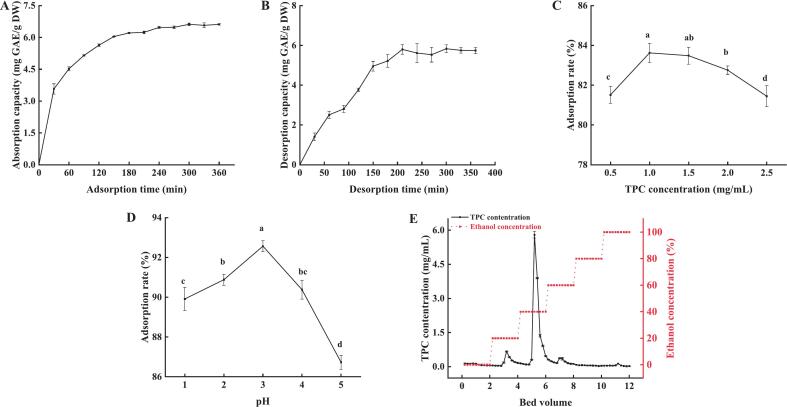
The static adsorption (A) and desorption (B) kinetics curves of D101 MAR for PPP. The effects of different concentrations (C) and pH (D) on the adsorption rate of D101 MAR. Dynamic desorption curves (E) at different ethanol concentrations. Results are presented as mean ± standard deviation (n = 3). Different lowercase letters in the line graph indicate significant differences (P < 0.05).

Adsorption kinetics is vital for understanding adsorbate attachment to an adsorbent, facilitating process optimization [50]. In the case of D101 MAR, the adsorption/desorption kinetics were evaluated at 25 °C to optimize purification time. The rapid adsorption of PPP by D101 MAR within the initial hour (Fig. 2A) could be attributed to pretreatment-enhanced active sites and surface area [46]. Subsequently, the adsorption rate slowered down when approaching saturation, mainly by mass transfer limitations [50]. The adsorption equilibrium was achieved during 180 min (6.21 mg GAE/g DW, 74.02 %). Desorption equilibrium was achieved during 210 min (5.79 mg GAE/g DW, 89.74 %) (Fig. 2B). The observations presented D101′s outstanding adsorption/desorption performance for the purification of PPP with excellent recovery efficiency.

In order to better elaborate the adsorption mechanisms and kinetics, PFO and PSO kinetic models were utilized to simulate the experimental data [49]. PFO model elucidated theinitial adsorption phase, while the PSO model canvased the entire process from a holistic perspective [51]. Table S3 depicted the kinetic equations and related parameters for PPP adsorption by D101 MAR, with the PSO model offering a higher correlation coefficient (R^2^ = 0.9956) than the PFO model (R^2^ = 0.9843), indicating superior process representation. Meanwhile, the PSO model's theoretical maximum adsorption capacity (Qe) nearly matched the experimental value (6.94 mg GAE/g DW), demonstrating the model's effectiveness in describing PPP adsorption onto D101 MAR. These findings aligned with Lu et al.'s [49] experiment, which also demonstrated the PSO model's effectiveness in polyphenol adsorption.

#### 3.2.3 Optimization of the adsorption conditions for D101 MAR

Fig. 2C illustrated the impact of the initial PPP concentration on the adsorption rate of D101 MAR. The adsorption rate initially increased, reaching a maximum of 83.63 % ± 0.39 % at a concentration of 1.0 mg/mL, then subsequently declined. The observed trend may be due to competitive binding at high concentrations, where impurities reduce polyphenol adsorption by competing for binding sites [52]. Accordingly, an initial concentration of 1.0 mg/mL was adopted for subsequent experiments.

Fig. 2D depicted pH's effect on D101 MAR adsorption efficiency. PPP adsorption rose from pH 1–3, reaching 92.56 % ± 0.22 % at pH 3, then decreased. This pattern stemmed from pH-dependent ionization of polyphenolic hydroxyl groups. Polyphenols in protonated form favored MAR adsorption at lower pH, though pH < 2 compromised resin structure and decreased adsorption capacity [53]. Therefore, pH 3 was selected as the optimal adsorption condition.

#### 3.2.4 Gradient elution of D101 MAR

A gradient ethanol elution (0 %, 20 %, 40 %, 60 %, 80 %, 100 %) was employed for the purification of PPP to eliminate impurities (Fig. 2E). Minimal PPP elution was observed at 0 % ethanol, with progressive elution starting at 20 % ethanol and reaching its peak at 40 % ethanol. Residual elution continued at 60 % ethanol, followed by negligible elution at higher concentrations. In PPP40, the recovery rate of polyphenols reached 90.01 % ± 1.24 % with a purity of up to 43.93 % ± 0.01 %, confirming significant enrichment efficiency (Table S4). This fraction exhibited minimal impurities after multiple cycles, making it optimal for subsequent analysis. Similar methodologies, as demonstrated by Jin et al., [54], had utilized gradient elution chromatography to isolate EGCG from green tea polyphenol extracts, effectively separating it from coextracted impurities.

### 3.3 Identification of PPP40

**Fig. 3:**
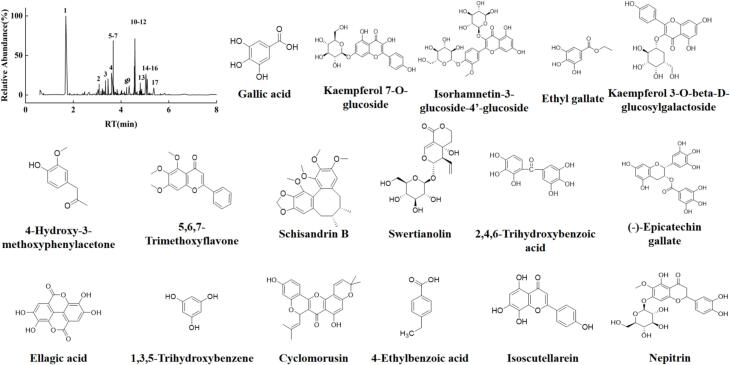
Total ion chromatograms and structural formulas of the major metabolites of PPP40.

The PPP40 extracts were subjected to accurate qualitative and quantitative analysis using UPLC–MS/MS, as depicted in the total ion chromatograms in Fig. 3. A total of 17 major phenolic compounds were identified through comparison of their retention times (Rt), mass spectrometry data (MS and MS/MS), and predicted molecular formulas (MF) with both published literature and the UNIFI database, encompassing phenolic acids, flavonoids, coumarins, and lignans. The detailed analytical parameters were detailed in Table 3. Among the identified compounds, the top eight based on response values gallic acid, kaempferol 7-*O*-glucoside, isorhamnetin-3-glucoside-4′-glucoside, ethyl gallate, kaempferol 3-*O*-beta-D-glucosylgalactoside, 4-hydroxy-3-methoxyphenylacetone, 5,6,7-trimethoxyflavone, and schisandrin B, with their structural formulas presented in Fig. 3.

**Table 3:** The identification of PPP40 by UPLC–MS/MS.

Serial Number	Name	Formular	RT/min	Adduct	Observed neutral mass /Da	Observed *m*/*z*	Mass error /ppm	Feature fragments	Response value
1	Gallic acid	C_7_H_6_O_5_	1.69	[M−H]^−^	170.0215	169.0153	2.70	125.0244, 107.0139, 79.0189	4,924,633
2	Kaempferol 7-*O*-glucoside	C_21_H_20_O_11_	4.58	[M+H]^+^	448.1006	449.1052	5.92	287.0559, 153.0182	3503,958
3	Isorhamnetin-3-glucoside-4′-glucoside	C_28_H_32_O_17_	3.67	[M+H]^+^	640.1639	641.1679	5.22	479.1184, 317.0656	3,381,123
4	Ethyl gallate	C_9_H_10_O_5_	4.56	[M+H]^+^	198.0528	199.0596	1.30	153.0546, 125.0233, 109.0284	1,795,857
5	Kaempferol 3-*O*-beta-D-glucosylgalactoside	C_27_H_30_O_16_	3.60	[M+H]^+^	610.1534	611.1580	4.40	449.1094, 287.0601	1,372,499
6	4-Hydroxy-3-methoxyphenylacetone	C_10_H_12_O_3_	5.04	[M+Na+HCOOH]^+^	180.0786	249.0749	3.93	153.0161, 105.0319, 77.0363	1,308,573
7	5,6,7-Trimethoxyflavone	C_18_H_16_O_5_	5.10	[M+K]^+^	312.0998	351.0698	17.08	298.0764, 283.0584, 199.0591	984,686
8	Schisandrin B	C_23_H_28_O_6_	3.35	[M+CH_3_CN+H]^+^	400.1886	442.2259	7.94	309.1168, 147.0636, 117.0895, 85.0271	901,510
9	Swertianolin	C_20_H_20_O_11_	4.81	[M−H]^−^	436.1006	435.0931	0.37	313.0552, 151.0039, 125.0245	749,137
10	2,4,6-Trihydroxybenzoic acid	C_7_H_6_O_5_	3.08	[M−H]^−^	170.0215	169.0148	1.45	125.0231, 107.0816	676,051
11	(−)-Epicatechin gallate	C_22_H_18_O_10_	5.06	[M+H]^+^	442.0900	443.093	9.70	273.0823, 159.0034	633,184
12	Ellagic acid	C_14_H_6_O_8_	4.34	[M−H]^−^	302.0063	300.9993	0.83	283.9957, 257.0078, 229.0136, 185.0231	569,742
13	1,3,5-Trihydroxybenzene	C_6_H_6_O_3_	4.56	[M+H]^+^	126.0317	127.0389	0.23	109.0259, 81.0317,	546,933
14	Cyclomorusin	C_25_H_22_O_6_	3.69	[M+H]^+^	418.1416	419.1527	8.97	351.0849, 349.0425, 167.0320	543,869
15	4-Ethylbenzoic acid	C_9_H_10_O_2_	4.24	[M+H]^+^	150.0681	151.0752	0.45	105.0681, 79.0530	485,325
16	Isoscutellarein	C_15_H_10_O_6_	5.37	[M+H]^+^	286.0477	287.0541	2.35	241.0477, 153.0167, 135.0425	443,291
17	Nepitrin	C_22_H_22_O_12_	3.66	[M+H]^+^	478.1111	479.1163	4.45	317.0640, 302.0393	426,654

**Fig. 4:**
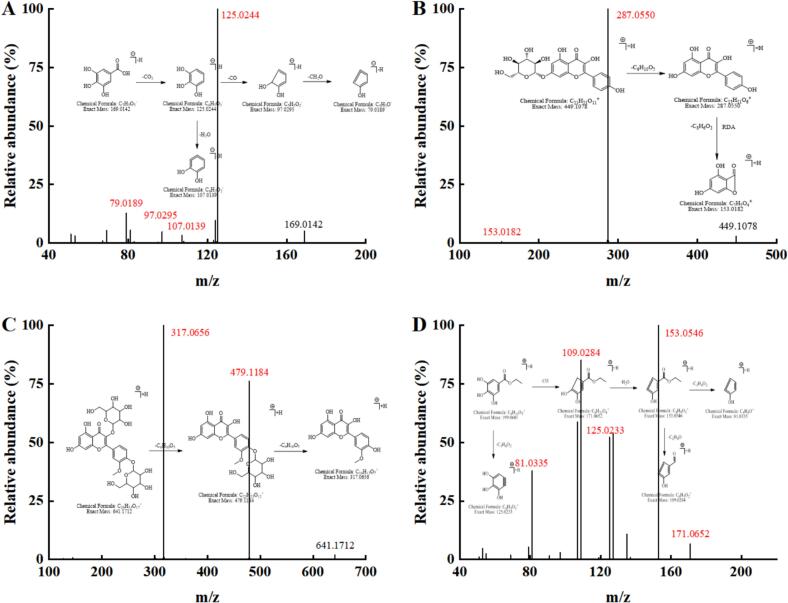
Secondary mass spectra and fragmentation pathway diagrams of gallic acid (A), kaempferol 7-*O*-glucoside (B), isorhamnetin-3-glucoside-4′-glucoside (C) and ethyl gallate (D).

Compound 1 exhibited an excimer ion peak at *m*/*z* 169.0153 in the negative ion [M−H]^−^ mode, corresponding to the elemental composition of C_7_H_5_O_5_^–^. The proposed fragmentation pathway of this compound, based on MS/MS analysis, was illustrated in Fig. 4A. M/z 169.0153 could obtain C_6_H_5_O_3_^–^ by losing one CO_2_, resulting in *m*/*z* 125.0244. Subsequent reverse Diels Alder reaction (RDA) opened the ring and released CO, yielding C_6_H_5_O_2_^–^ (*m*/*z* 97.0295) [55]. Further loss of one CH_2_O led to C_5_H_3_O^–^ (*m*/*z* 79.0189). Under another inference, *m*/*z* 125.0244 could directly lose one H_2_O molecule to obtain C_6_H_3_O_2_^–^, with *m*/*z* of 107.0139 [56]. This fragmentation pattern resembled the cleavage pathway of gallic acid as described by Medic et al. [57], confirming the identification of compound 1 as gallic acid based on UNIFI and previous studies.

Compound 2 exhibited a quasimolecular ion peak at [M+H]^+^ C_21_H_21_O_11_^+^ and *m*/*z* 449.1078 in positive ion mode. The MS/MS image and fragmentation pathway were presented in Fig. 4B. Initially, Compound 2 underwent the removal of a glucose group to yield C_15_H_11_O_6_^+^ (*m*/*z* 287.0550), a process that aligned with the cleavage mechanism outlined by Pan et al. [58] for the hydrolysis of kaempferol 7-*O*-glucoside to kaempferol monomer. Subsequently, the peak at *m*/*z* 287.0550 was further RDA cleaved, yielding C_7_H_5_O_4_^+^ with *m*/*z* 153.0182 [59]. Based on UNIFI and previous studies, kaempferol 7-*O*-glucoside was identified.

In positive ion mode, Compound 3 displayed a protonated molecular ion [M+H]^+^ at *m*/*z* 641.1712, corresponding to the molecular formula C_28_H_33_O_17_^+^. he MS/MS spectrum and proposed fragmentation pathway for Compound 3 were illustrated Fig. 4C. Compound 3 lost glucose group twice, resulting in C_22_H_23_O_12_^+^ (*m*/*z* 479.1184) and C_16_H_13_O_7_^+^ (*m*/*z* 317.0656) in sequence. This cleavage process conformed to the universal rule of gradual deglycosylation of diglycosides [60]. According to the information in the database, the substance was identified as isorhamnetin-3-glucoside-4′-glucoside.

Compound 4 exhibited an excimer ion peak at [M+H]^+^ C_9_H_11_O_5_^+^ and *m*/*z* 199.0601 in positive ion mode. The MS/MS image and fragmentation pathway were shown in Fig. 4D. Compound 4 might lose one C_3_H_6_O_2_ to obtain C_6_H_5_O_3_^+^ (*m*/*z* 125.0233) [61]. Alternatively, at *m*/*z* 199.0601, sequential losses of CO and one H_2_O result in C_8_H_9_O_3_^+^ (*m*/*z* 153.0546), followed by the loss of C_3_H_4_O_2_ or C_2_H_4_O to obtain C_5_H_5_O^+^ (*m*/*z* 81.0335) or C_6_H_5_O_2_^+^ (109.0284), respectively. According to the information in the database, the substance was identified as ethyl gallate [62], [63].

### 3.4 In vitro antioxidant activity

Plant phenolics exhibit antioxidant properties, linked to their potential in combating chronic diseases [64], [65]. The antioxidant potential of the extract from PPP40 was determined by DPPH and ABTS free radical scavenging test. DPPH assay only measures radical scavenging activity, while ABTS accurately measures the antioxidant activity of both hydrophilic and lipophilic substances [50].

**Fig. 5:**
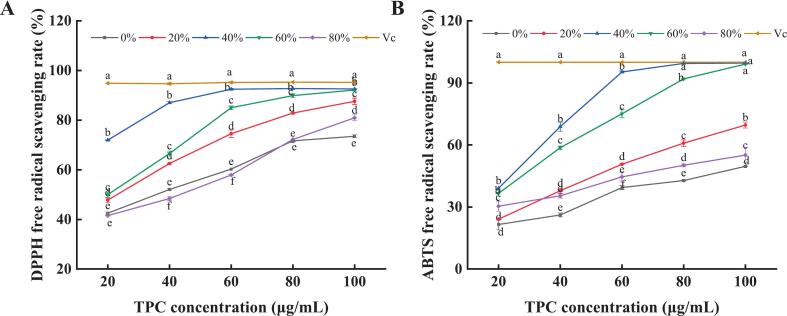
The scavenging ability of PPP40 against DPPH (A) and ABTS (B) radicals. Results are presented as mean ± standard deviation (n = 3). Different lowercase letters in the line graph indicate significant differences (P < 0.05).

Fig. 5A and B revealed dose-dependent DPPH and ABTS scavenging by PPP. The PPP40 exhibited superior scavenging activity across equivalent concentrations. Within the range of 20–100 μg/mL, DPPH scavenging increased from 71.95 % ± 0.07 % to 92.55 % ± 0.15 % (IC_50_ = 36.63 ± 2.00 μg/mL), while ABTS scavenging increased from 39.05 % ± 0.01 % to 99.58 % ± 0.12 % (IC_50_ = 40.12 ± 0.59 μg/mL). This superior activity can be analytically attributed to two key factors: (1) The high purity (43.93 %) and specific composition of PPP40, enriched in identified electron-donating phenolics like gallic acid and kaempferol glycosides, which effectively neutralize free radicals. (2) The integrated ultrasound-resin process, which likely preserved the structural integrity of these labile antioxidants more effectively than conventional methods. This observation aligns with the findings of Bai et al. [19], who reported potent DPPH and ABTS^+^ scavenging activities in peony pod extracts, further confirming the presence of antioxidant phenolics across Paeonia species. Overall, PPP40 represented an effective natural source of antioxidant.

### 3.5 Inhibitory effect and mechanism of PPP40 on α-glucosidase

#### 3.5.1 Inhibitory activity analysis

**Fig. 6:**
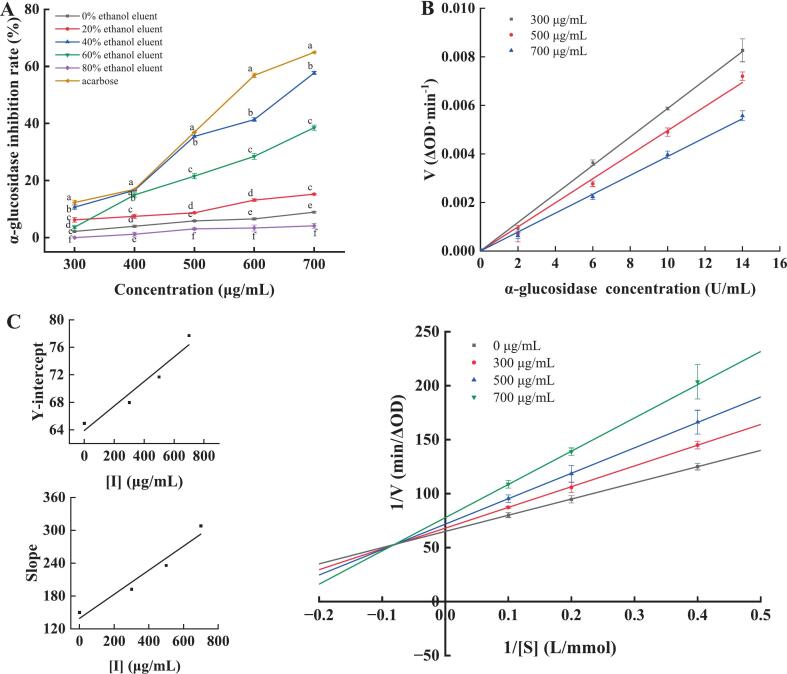
Inhibition curves of α-glucosidase by different concentrations of ethanol eluent and acarbose (A). Effect of PPP40 on α-glucosidase suppression dynamics (B) and Lineweaver–Burk (C) curves. Results are presented as mean ± standard deviation (n = 3). Different lowercase letters in the line graph indicate significant differences (P < 0.05).

Plant polyphenols inhibit α-glucosidase, offering potential treatment for post-meal hyperglycemia and type 2 diabetes [66]. Fig. 6A illustrated ethanol concentration-dependent α-glucosidase inhibition by PPP40 eluents, using acarbose as a positive control. All PPP fractions exhibited dose-dependent inhibition of α-glucosidase activity within the tested concentration range (300–700 μg/mL). Notably, PPP40 showed the strongest inhibition, correlating with its highest polyphenolic purity and antioxidant efficacy. At 700 μg/mL, acarbose showed 64.91 % ± 0.13 % inhibition (IC_50_ = 574.77 ± 7.40 μg/mL). Comparatively, the 40 % fraction achieved 57.69 % ± 0.50 % inhibition (IC_50_ = 639.96 ± 4.57 μg/mL), corresponding to 88.88 % acarbose efficacy. Similarly, Yemeni date polyphenols showed notable α-glucosidase inhibition *in vitro*, though weaker than acarbose [67]. PPP40 demonstrated significant α-glucosidase inhibitory activity *in vitro*, prompting further mechanistic studies to elucidate its mode of action.

#### 3.5.2 Inhibition kinetics analysis

The reversibility of PPP40-mediated α-glucosidase inhibition was assessed by plotting reaction rate (V) against enzyme concentration (Fig. 6B). Linear regression analysis revealed that all fitted lines intersected at the origin (R^2^ > 0.99). The slope magnitude decreased progressively with increasing PPP40 concentration, indicating that PPP40 impaired enzyme function by reducing its effectiveness in substrate degradation rather than by decreasing enzyme concentration [68]. This kinetic behavior suggested that PPP40 inhibits α-glucosidase activity through a reversible mechanism, where reversible inhibitors interacted with enzymes through noncovalent interactions, allowing for activity restoration through physical means [69].

To further characterize the enzyme inhibition kinetics and identify inhibition types and parameters, Lineweaver–Burk analysis was conducted [50]. In Fig. 6C, the regression lines converged in the second quadrant, and increasing PPP40 concentration resulted in an elevation of the K_m_ value from 2.31 to 3.96 while reducing V_max_ from 0.015 to 0.013, indicative of mixed inhibition [70]. Similar Lineweaver–Burk plots were observed with buckwheat hull polyphenols [34] and sugarcane polyphenol extracts [71], confirming mixed-type α-glucosidase inhibition. Simultaneously, the competitive (K_i_) and uncompetitive (K_is_) inhibition constants were determined through analysis of Lineweaver–Burk plots for PPP40, yielding K_i_ = 0.63 mg/mL and K_is_ = 3.59 mg/mL, where lower values corresponded to greater inhibitory potency against α-glucosidase. The lower K_i_ versus K_is_ indicated PPP40 preferentially bound to free α-glucosidase over the enzyme–PNPG complex [72]. In conclusion, PPP40 inhibited α-glucosidase reversibly via a mixed-type mechanism.

#### 3.5.3 Fluorescence quenching analysis

**Fig. 7:**
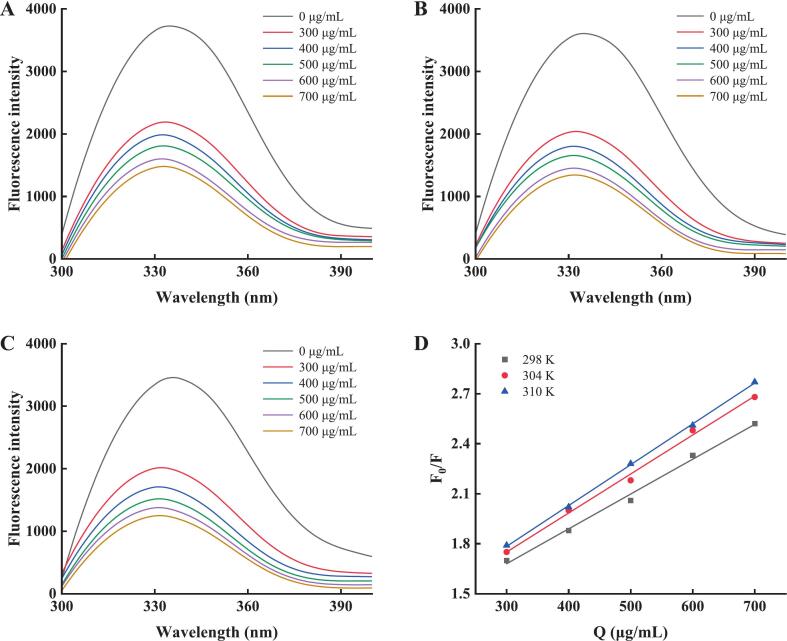
Fluorescence quenching spectra of PPP40 toward α-glucosidase at 298 K (A), 304 K (B), and 310 K (C) and Stern–Volmer plots (D) of PPP40 with α-glucosidase.

Polyphenol binding to α-glucosidase quenches fluorescence via interactions with tryptophan/tyrosine residues through hydrogen bonding and hydrophobic interactions, reducing fluorescence intensity [73]. Experiments were conducted at 298 K, 304 K, and 310 K to assess temperature effects. The choice of 298 K aimed to maintain methodological consistency, 310 K simulated physiological conditions, and 304 K bridged thermal gradients for robust thermodynamic analysis [12]. Fig. 7A–C illustrated PPP40-induced fluorescence quenching of α-glucosidase across different temperatures. The extent of quenching indicates significant PPP40–α-glucosidase chromophore interactions. A consistent blue-shift in emission maxima occurred across all temperatures, suggesting that PPP40 binding induced conformational changes in the enzyme through hydrogen bonding and hydrophobic interactions, leading to the burial of residues into hydrophobic pockets and a reduction in local polarity [71].

Fluorescence quenching mechanisms were classified as static or dynamic quenching [74], [75]. The quenching mechanism was elucidated through Stern–Volmer analysis, as depicted in Fig. 7D. Linear Stern–Volmer plots (R^2^ > 0.99) at varying temperatures confirmed a consistent quenching mechanism for the interactions between PPP40 and the enzyme. As shown in Table 4, the rising Stern–Volmer quenching constant (K_SV_) values with higher temperatures indicated dynamic quenching, where increased molecular interactions at elevated temperatures enhanced the quenching constant [76]. Cheng et al., [77] reported a similar fluorescence quenching mechanism in their study of the inhibition of starch-digesting enzymes by ferulic acid and quercetin.

**Table 4:** Fluorescence quenching constants and thermodynamic parameters of PPP40 for α-glucosidase.

Temperature (K)	K_SV_ (mL·μg^−1^)	K_q_ × 10^7^ (mL·μg^−1^·s^−1^)	ΔH (kJ·μg^−1^)	ΔS (J·μg^−1^·K^−1^)	ΔG (kJ·μg^−1^)
298	1.52	1.52	8.29	31.28	−1.03
304	1.68	1.68			−1.22
310	1.77	1.77			−1.41

Biomolecular binding involves four primary forces: electrostatic, hydrophobic, van der Waals, and hydrogen bonding interactions [78]. Thermodynamic parameters were employed to characterize the types of interactions between α-glucosidase and PPP40. In Table 4, positive values were observed for ΔH (8.29 kJ·μg^−1^) and ΔS (31.28 J·μg^−1^·K^−1^) in the PPP40–α-glucosidase system. The positive values of both enthalpy change (ΔH) and entropy change (ΔS) point towards hydrophobic forces as the predominant driving force, indicating that PPP40 predominantly interacted with α-glucosidase through hydrophobic interactions. While ΔG < 0 indicated that the binding of PPP40 to α-glucosidase occurs spontaneously and releases heat [79]. In summary, the interaction of PPP40 with α-glucosidase occurred through a spontaneous, exothermic process, predominantly driven by dynamic fluorescence quenching mediated by hydrophobic interactions, leading to conformational changes in the enzyme.

#### 3.5.4 CD spectra analysis

**Fig. 8:**
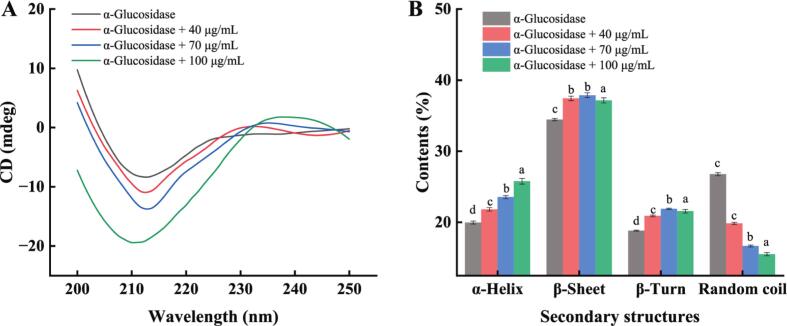
CD spectroscopy of α-glucosidase at different concentrations of PPP40 (A) and secondary structure content diagram (B). Dynamic desorption curves at different ethanol concentrations. Results are presented as mean ± standard deviation (n = 3). Different lowercase letters in the column chart indicate significant differences (P < 0.05).

CD provided valuable insights into protein secondary structure by detecting conformational changes in the polypeptide backbone within the far–ultraviolet region spectrum [72]. Fig. 8 depicted alterations in α-glucosidase's secondary structure due to differing PPP40 levels. Increasing PPP40 concentration induced structural changes in α-glucosidase: α-helix content increased by 5.70 % ± 0.24 % (20.37 % ± 0.31 % to 26.07 % ± 0.49 %), β-sheet content increased by 2.43 % ± 0.29 % (35.13 % ± 0.15 % to 37.57 % ± 0.32 %), β-turn content increased by 2.60 % ± 0.16 % (19.20 % ± 0.20 % to 21.80 % ± 0.20 %), and random coil content decreased by 11.60 % ± 0.41 % (27.30 % ± 0.26 % to 15.70 % ± 0.26 %). The transition from random coil to more ordered secondary structures, indicated an improvement in the structural stability of α-glucosidase. This stabilization was likely facilitated by the insertion of PPP40 into hydrophobic regions on the enzyme surface, leading to a segmental reorganization of the protein chain and perturbation of specific regional structures, thereby enhancing noncovalent interactions (hydrogen bonding, hydrophobic interactions) [80], [81]. These CD-derived insights into binding-induced stabilization were consistent with and provide a mechanistic explanation for the mixed-type inhibition kinetics and dynamic fluorescence quenching observed previously. Collectively, these findings suggested a mechanism whereby PPP40 restricts the enzyme's functional dynamics through non-covalent interactions.

## 4 Conclusions

This study developed an efficient integrated process for the extraction and purification of the polyphenol-rich fraction PPP40 from peony pods, an underutilized agricultural by-product. Comprehensive characterization by UPLC-MS/MS identified 17 major phenolic constituents, with gallic acid, kaempferol 7-*O*-glucoside, isorhamnetin-3-glucoside-4′-glucoside, and ethyl gallate as principal components. The obtained PPP40 fraction exhibited significant *in vitro* antioxidant capacity and effectively inhibited α-glucosidase activity. Kinetic analysis revealed that PPP40 acted as a mixed-type and reversible inhibitor of α-glucosidase. Fluorescence quenching studies demonstrated that PPP40 quenched enzyme fluorescence through a dynamic mechanism. CD spectroscopy indicated that PPP40 induced conformational structural alterations in the secondary conformation of α-glucosidase, disrupting its hydrophobic interactions leading to diminished enzymatic function.

This work established the ultrasound-integrated process as a viable foundation for the efficient production of PPP40 and highlighted its potential as a natural α-glucosidase inhibitor. These *in vitro* findings strongly support the anti-diabetic potential of PPP40. However, current findings are based solely on *in vitro* experiments, future work should focus on elucidating its systemic biological targets through network pharmacology and validating its efficacy and safety in cellular and animal models.

## CRediT authorship contribution statement

**Xiaoai Zhu:** Writing – original draft, Project administration, Funding acquisition, Conceptualization. **Yuan Ru:** Writing – original draft, Methodology, Investigation, Data curation. **Kebing Yan:** Methodology, Formal analysis, Data curation. **Siqi Zhang:** Methodology, Investigation. **Yingjie Sun:** Methodology, Formal analysis. **Fengke Du:** Formal analysis, Data curation. **Yage Liu:** Methodology, Investigation. **Yiying Niu:** Investigation, Formal analysis. **Jun Xi:** Writing – review & editing, Supervision. **Kunlun Liu:** Writing – review & editing, Project administration.

## Declaration of competing interest

The authors declare that they have no known competing financial interests or personal relationships that could have appeared to influence the work reported in this paper.
