# Supplementary material for: Ultrasound-assisted and resin-based purification of bioactive polyphenols from peony (Paeonia ostii) pods: process optimization and α-glucosidase inhibitory activity
Source: Ultrason Sonochem. 2026 Feb 12;127:107775. doi: 10.1016/j.ultsonch.2026.107775 (PMC12925347; doi:10.1016/j.ultsonch.2026.107775)
Supplement: Supplementary Data 1 [file mmc1.docx]

**Table S1**

Independent variables of Box–Behnken Design experiment design.

| **Level of value** | **A** | **B** | **C** |
| --- | --- | --- | --- |
|  | **Ethanol concentration (%)** | **Liquid–solid ratio (mL/g)** | **Extraction time (min)** |
| −1 | 30 | 5 | 20 |
| 0 | 45 | 10 | 40 |
| 1 | 60 | 15 | 60 |

**Table S2**

Experimental conditions based on Box–Behnken design and the corresponding responses.

| **Runs** | **Coded (Independent variables)** | | | **Yield（mg GAE/g DW）** |
| --- | --- | --- | --- | --- |
|  | **A** | **B** | **C** |  |
| 1 | −1 | −1 | 0 | 19.95 ± 0.38 |
| 2 | 1 | −1 | 0 | 28.45 ± 0.19 |
| 3 | −1 | 1 | 0 | 38.45 ± 0.86 |
| 4 | 1 | 1 | 0 | 41.85 ± 0.54 |
| 5 | −1 | 0 | −1 | 32.15 ± 0.30 |
| 6 | 1 | 0 | −1 | 40.85 ± 0.18 |
| 7 | −1 | 0 | 1 | 37.78 ± 0.10 |
| 8 | 1 | 0 | 1 | 42.45 ± 0.41 |
| 9 | 0 | −1 | −1 | 30.85 ± 0.20 |
| 10 | 0 | 1 | −1 | 45.25 ± 0.47 |
| 11 | 0 | −1 | 1 | 33.78 ± 0.04 |
| 12 | 0 | 1 | 1 | 48.90 ± 0.05 |
| 13 | 0 | 0 | 0 | 44.50 ± 0.29 |
| 14 | 0 | 0 | 0 | 43.05 ± 0.48 |
| 15 | 0 | 0 | 0 | 44.25 ± 0.68 |
| 16 | 0 | 0 | 0 | 43.92 ± 0.44 |
| 17 | 0 | 0 | 0 | 42.85 ± 0.35 |

**Table S3**

Adsorption kinetics equations and parameters of PPP adsorption onto D101 MAR.

| **Models** | **Equations** | **R^2^** | **Parameters** |
| --- | --- | --- | --- |
| Pseudo-first order | Ln (Q_e_–Q_t_)=–0.0115t+1.4415 | 0.9843 | Q_e_=4.22 mg/g; k_1_=0.0115 |
| Pseudo-second order | $\frac{\text{t}}{\text{Q}_{\text{t}}}$=0.1441t+3.0588 | 0.9956 | Q_e_=6.94 mg/g; k_2_=0.0068 |

**Table S4**

Recovery rate and purity of PPP elution solutions with different ethanol concentrations.

| **Ethanol concentration (%)** | **Recovery rate (%)** | **Purity (%)** |
| --- | --- | --- |
| 0 | 6.60 ± 0.01 % | 6.71 ± 0.02 % |
| 20 | 14.05 ± 0.27 % | 7.91 ± 0.01 % |
| 40 | 90.01 ± 1.24 % | 43.93 ± 0.01 % |
| 60 | 15.07 ± 0.30 % | 16.97 ± 0.01 % |
| 80 | 3.61 ± 0.01 % | 3.83 ± 0.01 % |
| 100 | 3.24 ± 0.01 % | 3.43 ± 0.01 % |
